# MiR-1292 Targets FZD4 to Regulate Senescence and Osteogenic Differentiation of Stem Cells in TE/SJ/Mesenchymal Tissue System via the Wnt/β-catenin Pathway

**DOI:** 10.14336/AD.2018.1110

**Published:** 2018-12-04

**Authors:** Junfen Fan, Xingyan An, Yanlei Yang, Haoying Xu, Linyuan Fan, Luchan Deng, Tao Li, Xisheng Weng, Jianmin Zhang, Robert Chunhua Zhao

**Affiliations:** ^1^Institute of Basic Medical Sciences Chinese Academy of Medical Sciences, School of Basic Medicine Peking Union Medical College, Center of Excellence in Tissue Engineering Chinese Academy of Medical Sciences, Beijing Key Laboratory (No. BZO381), Beijing, China.; ^2^Department of Orthopaedic Surgery, Peking Union Medical College Hospital, Peking Union Medical College, Beijing, China.; ^3^Department of Immunology, Research Center on Pediatric Development and Diseases, Institute of Basic Medical Sciences Chinese Academy of Medical Sciences and School of Basic Medicine Peking Union Medical College, State Key Laboratory of Medical Molecular Biology, Beijing, China

**Keywords:** TE/SJ/mesenchymal tissue system, miR-1292, hADSCs, senescence, osteogenesis, FZD4

## Abstract

With the expansion of the elderly population, age-related osteoporosis and the resulting bone loss have become a significant health and socioeconomic issue. In Triple Energizer (TE)/San Jiao (SJ)/mesenchymal tissue system, mesenchymal stem cell (MSC) senescence, and impaired osteogenesis are thought to contribute to age-related diseases such as osteoporosis. Therefore, comprehending the molecular mechanisms underlying MSC senescence and osteogenesis is essential to improve the treatment of bone metabolic diseases. With the increasing role of miRNAs in MSC aging and osteogenic differentiation, we need to understand further how miRNAs participate in relevant mechanisms. In this study, we observed that the expression of miR-1292 was augmented during cellular senescence and lessened with osteogenesis in human adipose-derived mesenchymal stem cells (hADSCs). miR-1292 expression was positively correlated with senescence markers and negatively associated with bone formation markers in clinical bone samples. Overexpression of miR-1292 notably accelerated hADSC senescence and restrained osteogenesis, whereas its knockdown decreased senescence and enhanced osteogenic differentiation. Furthermore, miR-1292 upregulation inhibited ectopic bone formation *in vivo*. Mechanistically, FZD4 was identified as a potential target of miR-1292. Downregulation of FZD4 phenocopied the effect of miR-1292 overexpression on hADSC senescence and osteogenic differentiation. Moreover, the impact of miR-1292 suppression on senescence and osteogenesis were reversed by the FZD4 knockdown. Pathway analysis revealed that miR-1292 regulates hADSC senescence and osteogenesis through the Wnt/β-catenin signaling pathway. Thus, TE/SJ/mesenchymal tissue system is the largest organ composed of various functional cells derived from mesoderm, responsible for maintaining homeostasis and regulating cell senescence. miR-1292 might serve as a novel therapeutic target for the prevention and treatment of osteoporosis or other diseases related to bone metabolism and aging.

In traditional Chinese medicine (TCM), one fundamental yet essential concept is the Triple Energizer (TE) or San Jiao (SJ) which states that ‘the upper, middle and lower San Jiao connect to be one Qi, protecting human body’, and that the ‘San Jiao is not a true Fu without structure but having a function’. With the development of the state of art science and technology, especially cell biology and medical biopsy approaches, scientists have done more extensive research on the human body in recent years than ever before. For more than 20 years, we have investigated the characteristics of mesenchymal stem cells (MSCs) and began to explore the concepts of TE/SJ and MSC system. Recently, we have isolated MSC/pericytes, the primary functional cells of TE/SJ structure and identified that pericytes separated from living brain tissues have contractile function [1]. We propose that TE/SJ is the largest organ consisting of all functional cells derived from the mesoderm during embryonic development. There is also a special fluid-filled chamber surrounded by mesothelium links to other vital organs. TE/SJ/mesenchymal tissue system plays a pivotal role in stem cell storage, water metabolism, nutrient transport, immune regulation, hormone transport, signal transduction, and tissue homeostasis. Therefore, TE/SJ/mesenchymal tissue system is involved in the stem cell proliferation, differentiation, senescence, and immune surveillance, as well as regulation of tissue regeneration and metabolism balance.

Aging is a biological process that advances with the gradual deterioration of functionality in living organisms. Osteoporosis is one of the most common orthopedic problems in aged populations [2]. With its high incidence and detrimental effects, osteoporosis is becoming an ‘invisible killer’ worldwide. Throughout life, bone is maintained in a dynamic balance through the processes of bone formation by osteoblasts and bone resorption by osteoclasts [3]. However, in many individuals, bone mass homeostasis begins to fail in the middle age, leading to bone loss, osteoporosis, and fragility-linked fractures [4,5]. We believe that the essence of aging involves the loss of TE/SJ stem cells and their secretion of regulatory factors. Therefore, we attempted to study the mechanism of aging by using stem cell senescence model in the mesenchymal tissue system. MSCs are progenitor cells of osteoblasts, which are involved in bone formation. MSC senescence is a facilitative factor of aging and aging-related diseases [6-8]. Accordingly, it is thought that a reduction in the differentiation of MSCs into osteoblasts contributes to the impaired bone formation observed during osteoporosis [9,10]. To date, in-depth understanding of the mechanisms involved in MSC senescence and osteogenesis is lacking [11]. Thus, elucidating the molecular processes that control MSC senescence and osteogenesis is vital to develop valid therapies for osteoporosis and other age-associated bone metabolic diseases.

Numerous transcription factors and various extracellular and intracellular signals can regulate cellular senescence and osteogenesis, such as p53/p21, p16/RB, Wnt/β-catenin, TGF-β/BMPs/Smads, Notch, and MAPK pathways [12-18]. Nevertheless, how the specific factors implicated in this process orchestrate to regulate the senescence and osteogenic differentiation of MSCs remain elusive. miRNAs are small (~22 nucleotides), single-stranded non-coding RNAs that are extensively involved in regulating target genes at the post-transcriptional level. One miRNA can regulate hundreds of different target genes, and one coding gene might be regulated by multiple miRNAs [19]. miRNAs are recognized as important regulators of various biological processes, including cell proliferation, migration, differentiation, apoptosis, and tissue development [20-22]. Furthermore, the role of miRNAs in MSC senescence and osteogenic differentiation is emerging [23-25]. Targeting miRNAs has been suggested as a new therapeutic approach for several diseases such as osteoporosis, HCV-related diseases, and colorectal cancer [26-28]. A novel miRNA, miR-1292, located on chromosome 20p13, has only been reported to be dysregulated in patients with systolic heart failure [29] and has a pro-metastatic role in colorectal cancer progression and metastasis [30]. However, its role in the regulation of MSC senescence and osteogenic differentiation, as well as potential associated mechanisms, has not been elucidated.

In this study, we demonstrate that miR-1292 is positively linked with senescence-associated factors and negatively associated with bone formation markers in clinical bone samples. Specifically, miR-1292 was found to enhance hADSC senescence and suppress osteogenic differentiation *in vitro* and delay bone formation *in vivo* by targeting FZD4 via the Wnt/β-catenin pathway. These findings suggest that inhibiting miR-1292 could delay senescence and enhance bone formation. Thus, miR-1292/FZD4 might serve as a novel therapeutic target for the prevention and treatment of osteoporosis and other age-associated bone diseases.

## MATERIALS AND METHODS

### hADSC isolation and culture

The hADSCs were isolated and cultured as previously described [31]. The Ethics Committee of the Chinese Academy of Medical Sciences and Peking Union Medical College approved all procedures performed in this study. Briefly, cells were isolated from adipose tissue and cultured in DMEM/F-12 supplemented with 2% fetal bovine serum (FBS; Gibco, USA), 1 x Insulin-Transferrin-Selenium (ITS; Gibco, USA), 10 ng/mL EGF (Peprotech, USA), 10 ng/mL PDGF (Peprotech, USA), 50 μM β-mercaptoethanol (Sigma, USA), 2 mM L-glutamine (Invitrogen, USA), 100 U/mL penicillin and 100 μg/mL streptomycin. The cells were maintained at 37 °C in a humidified incubator with 5% CO_2_. We used different concentrations (10, 20 or 40 μM) of XAV939 (Selleckchem, USA) to examine the effects of the Wnt/β-catenin pathway on cellular senescence and osteogenic differentiation.

### Senescence-associated β-galactosidase (SA-β-gal) staining

A Senescence β-Galactosidase Staining Kit was employed to measure the activity of SA-β-gal in hADSCs from different passages (Yeasen, Shanghai, China) according to the manufacturer’s instructions. Briefly, cells were washed twice with PBS and fixed with 4% paraformaldehyde for 15 min. Next, cells underwent washing in PBS followed by incubation with SA-β-gal staining solution at 37 °C in the dark for 24 h. The positive cells stained blue, and the images were acquired using an inverted microscope (Olympus, Japan).

### Clinical bone sample preparation

The Orthopedic Department of Peking Union Medical College Hospital provided seventy clinical bone specimens for this study. The samples were from patients who had a fracture from falling but without apparent violence. The additional exclusions comprised individuals with malignancy, diabetes, or other severe diseases over the past five years. The Ethics Committee of the Chinese Academy of Medical Sciences and Peking Union Medical College approved all clinical procedures.

### Osteoblast differentiation of hADSCs

When cells (2 x 10^5^) plated onto 6-well plates reached ~80% confluence, the growth medium was changed to osteoblast induction medium containing DMEM supplemented with 10% FBS, 10 mM β-glycerophosphate (Sigma, USA), 0.5 mM L-ascorbic acid (Sigma, USA), and 0.01 mM dexamethasone (Sigma, USA).

### Alkaline phosphatase (ALP) and alizarin red staining

ALP staining was performed using an ALP staining kit (Institute of Hematology and Blood Diseases Hospital, Chinese Academy of Medical Sciences, Tianjin, China) according to the manufacturer’s protocol on days 4 and 6 of osteoblast differentiation. Alizarin red staining was conducted to detect matrix mineralization deposition on days 12 and 15 after the initiation of differentiation. In brief, cells were washed twice with PBS, fixed with 4% paraformaldehyde for 10 min, rinsed with double-distilled H_2_O, and stained with 1% alizarin red (pH 4.2; Leagene, Beijing, China) staining solution for 30 min at room temperature. The cells were photographed following a thorough wash in double-distilled H_2_O to remove the unbound dye.

### ALP activity assay

The cells were washed twice with cold PBS and lysed with radioimmunoprecipitation (RIPA) lysis buffer (Beyotime, Shanghai, China). After centrifugation, 3-5 μL of cell supernatant was incubated with 200 μL of the Alkaline Phosphatase Yellow Liquid Substrate System (pNPP) reagent (Sigma, USA) at 37 °C for 30 min. The reaction was determined using a spectrophotometer at 405 nm following blocking with 50 μL of 3 M NaOH. ALP activity was normalized to total protein in the cell lysates.

### miRNA agomir, antagomir, and siRNA transfection

We employed Lipofectamine 2000 (Invitrogen, USA) as per manufacturer’s instructions to transfect cells with miR-1292 agomir, antagomir, siRNA, or related negative controls (NCs). The miR-1292 agomir, antagomir, and negative controls were synthesized by GenePharma (Shanghai, China). FZD4 specific siRNAs were obtained from Genebio (Shanghai, China). Sequences are listed in Supplementary Table 1.

### RNA extraction and qRT-PCR analysis

Total RNA was extracted from cultured cells or fresh bone tissues with TRIzol reagent (Invitrogen, USA) according to the manufacturer’s protocol and treated with DNase I (Ambion, USA) at 37 °C for 30 min. Reverse transcription was performed using a Reverse Transcription kit (Takara, Japan) according to the manufacturer’s instructions. qRT-PCR was performed with Hieff^TM ^qPCR SYBR^®^ Green Master Mix (Yeasen, Shanghai, China) using a Step One Plus Real-Time PCR Detection System (Applied Biosystems, USA). Relative expression of mRNA or miRNA was evaluated by the 2^-ΔΔCt^ method and normalized to the expression of *GAPDH*, *PPIA* [32], or *U6*, respectively. The supplementary Table 1 shows the primers employed for amplification of these genes.

### Western blot analysis

Total protein of cells was lysed with RIPA lysis buffer containing a protease inhibitor cocktail (Beyotime, China) and quantified with a BCA Protein Assay kit (Beyotime, China). Protein fractions were separated by sodium dodecyl sulfate-polyacrylamide electrophoresis (SDS-PAGE) gels, and then transferred to 0.45 µm PVDF membranes (Millipore, USA). The membranes were blocked with 5% non-fat milk in TBST for 1 h and then incubated with specific primary antibodies at 4 °C overnight, followed by incubation with secondary antibodies for 1 h. Bands were visualized using a chemiluminescence ECL reagent (Millipore, USA).

**Figure 1.**
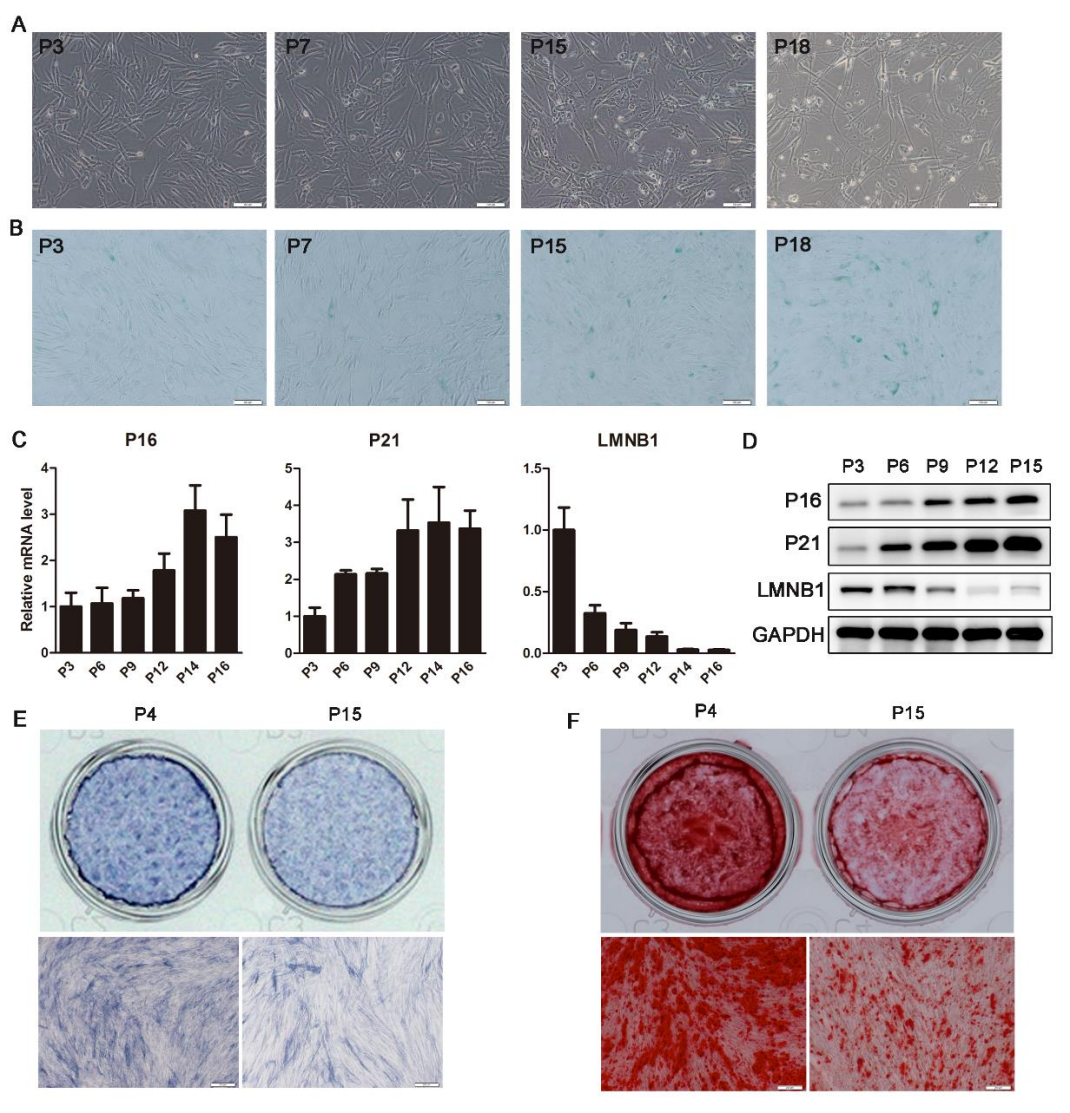
Characterization of senescent hADSCs. (A) The morphology of hADSCs, as observed under the microscope at passage 3, 7, 15, and 18 (P3, 7, 15, and 18). (B) SA-β-gal staining (blue) of hADSCs at P3, 7, 15, and 18. (C) qRT-PCR demonstrating mRNA expression levels of P16, P21, and LMNB1 at different passages of hADSCs. (D) Western blot analysis of protein levels of P16, P21, and LMNB1 at different passages of hADSCs. (E) ALP staining was performed on day 4 using different hADSC passage numbers (P4 and P15). (F) Alizarin red staining was performed to indicate mineral deposition on day 15 of different hADSC passages (P4 and P15). GAPDH was used as an internal control for western blot analysis, whereas qRT-PCR data were normalized to *PPIA*; results are presented as the mean ± SD, n = 3. Scale bars: 100 μm (A and B) or 200 μm (E and F).

### Ectopic bone formation in vivo

Cells were loaded on hydroxyapatite/tricalcium phosphate (HA/TCP) ceramic powder (National Engineering Research Center for Biomaterials, Chengdu, China), and implanted subcutaneously into the dorsal surface of 8-week-old NOD/SCID mice as previously described [33,34]. Briefly, 2×10^6^ cells were suspended in 200 μL of medium, mixed with 80 mg of wet HA/TCP, incubated at 37 °C overnight, and implanted into non-obese diabetic/severe combined immunodeficiency (NOD/SCID) mice. The implants were removed after 8 weeks, fixed in 4% paraformaldehyde, decalcified in 10% EDTA and embedded in paraffin. Staining of thin sections (5 μm) comprised hematoxylin-eosin (H&E), Masson trichrome, and immunohistochemical methods using specific antibodies. All animal experiments were performed per guidelines and approvals by the Ethics Committee of the Chinese Academy of Medical Sciences and Peking Union Medical College.

### Dual luciferase reporter assay

Approximately 90 bp synthetic fragments comprising *NFAT5*, *GDNF*, *FZD4*, *FZD5*, and *CLCN5* 3′ UTRs, which contained the miR-1292 predicted seed match site or a mutant site, were inserted between the Not I and Xho I cleavage sites of the psiCHECK-2 vector (Promega, USA). The supplementary Table 2 shows the sequences of relative fragments employed. HEK293T cells were co-transfected with the luciferase reporter vector and miR-1292 agomir or miR-NC using Lipofectamine 2000. *Renilla* and *firefly* luciferase activities were measured in triplicate 48 h after transfection by the Dual Luciferase Reporter Assay System using the manufacturer’s instructions (Promega, USA).

**Figure 2.**
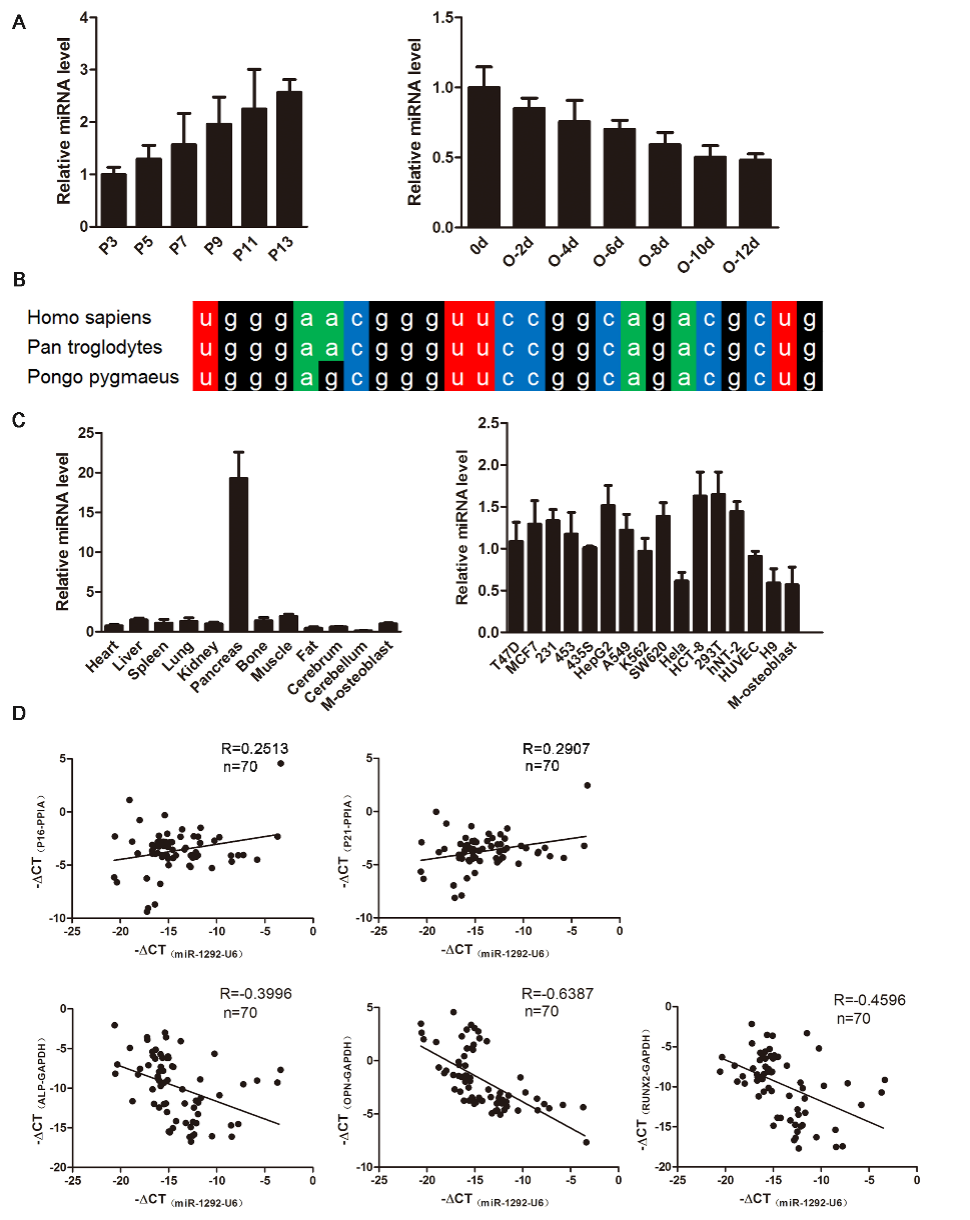
Expression analysis of miR-1292. (A) qRT-PCR analysis of the dynamic expression of miR-1292 during the senescence and osteogenic differentiation of hADSCs. (B) Conservation of miR-1292 in mammals. (C) Expression of miR-1292 was evaluated in mouse tissues and various cell lines (M-osteoblast: hADSC-derived osteoblast) by miRNA-specific qRT-PCR. (D) The correlations between the expression of senescence markers and miR-1292 or bone formation markers and miR-1292 in clinical bone samples were detected by qRT-PCR. The data, normalized to U6, PPIA or GAPDH expression, are presented as the mean ± SD, n = 3.

### Statistical analysis

The data were analyzed using GraphPad Prism 5 software (GraphPad Software Inc.) and presented as the mean ± SD. Statistical comparisons between two groups utilized Student’s t-tests, whereas comparisons of multiple groups used one-way ANOVA. Differences were considered statistically significant at ^*^P < 0.05, ^**^P < 0.01 and ^***^P < 0.001.

**Figure 3.**
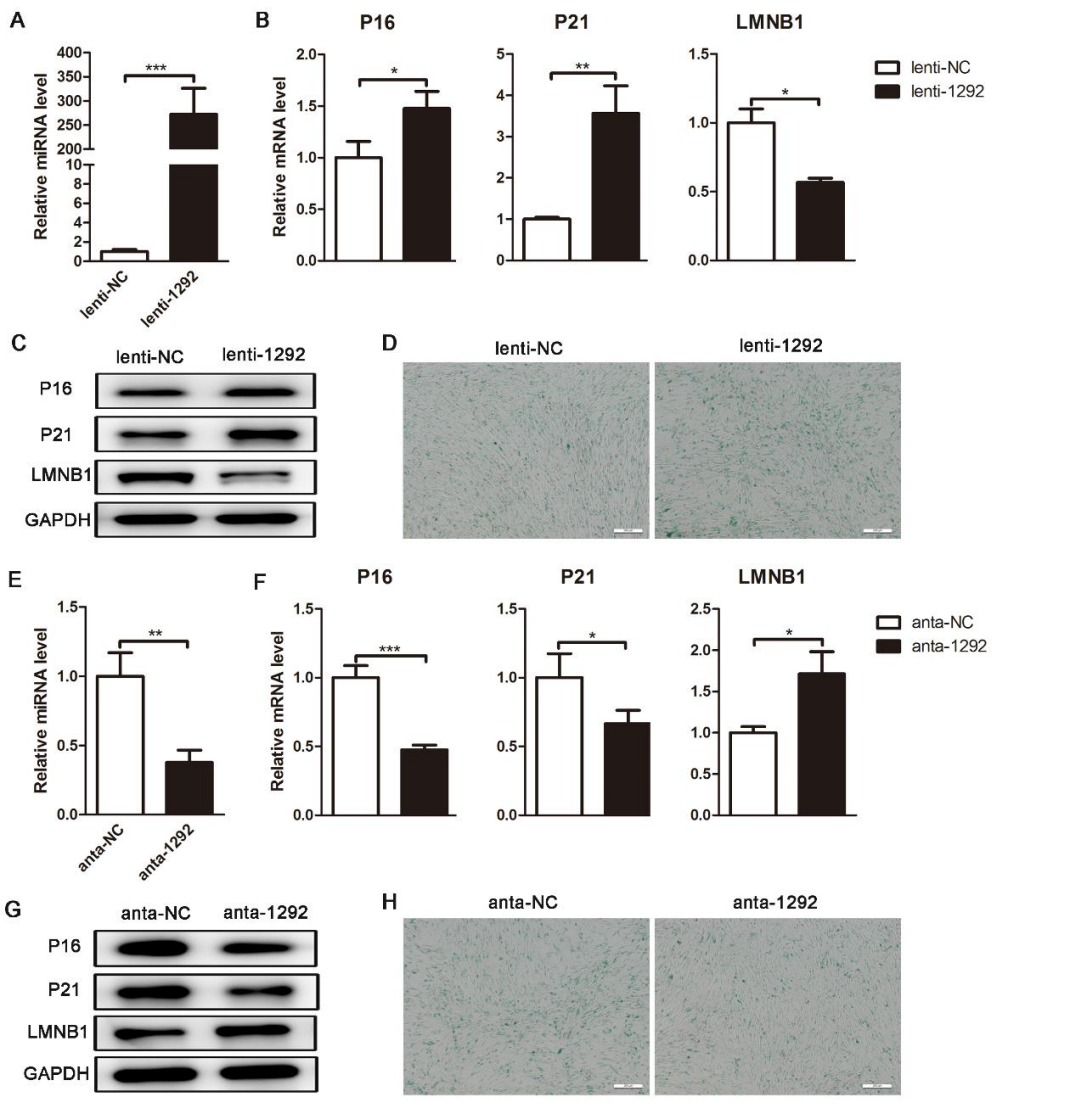
miR-1292 regulates hADSC senescence. (A) miR-1292 expression was evaluated by qRT-PCR after lenti-1292 treatment. (B and C) qRT-PCR and western blot were performed to analyze the mRNA and protein levels of P16, P21, and LMNB1 after miR-1292 overexpression. (D) SA-β-gal staining was performed to detect the activity of SA-β-gal after miR-1292 overexpression. (E) Stem-loop RT-PCR determined miR-1292 expression after anta-1292 transfection. (F and G) The expression levels of P16, P21, and LMNB1 were analyzed by qRT-PCR and western blot after inhibiting miR-1292. (H) SA-β-gal staining was performed after inhibiting miR-1292. GAPDH was used as an internal control for western blot analysis. The quantitative data normalized to U6 or PPIA, are presented as the mean ± SD, n = 3. ^*^P < 0.05, ^**^P < 0.01, ^***^P < 0.001 compared to the control. Scale bars: 200 μm.

## RESULTS

### Characterization and validation of senescent hADSCs

Through the repeated subculture of hADSCs, we established a cell model of replicative senescence *in vitro*. During the senescent stage, cells underwent irreversible growth arrest and displayed a combination of senescence-related phenotypes. Specifically, after receiving 15 trypsin passages from the primary cells (P15), hADSCs gradually became larger with a flat and irregular shape (Fig. 1A), which is the typical morphology of senescent cells. Further, SA-β-gal secretion increased after P15 (Fig. 1B) and mRNA and protein levels of cell senescent molecular markers, specifically P16 and P21, were significantly upregulated in cultured MSCs from P3 to P16, whereas lamin B1 (LMNB1) levels were dramatically decreased (Fig. 1C, D). To compare the osteogenic differentiation potential during cell senescence, hADSCs of P4 and P15 were cultured in osteogenic induction medium and assessed by ALP staining at day 4 and alizarin red staining at day 15. As shown in Figure 1E, the expression of ALP, an early marker of osteoblasts, was decreased in P15 cells as compared to that in P4 hADSCs. Calcium salt formation was also markedly diminished in hADSCs at P15 compared to that at P4 (Fig. 1F). Thus, when compared to earlier passages, late passage hADSCs (P15 or later) displayed a senescent phenotype and decreased osteogenic potential.

**Figure 4.**
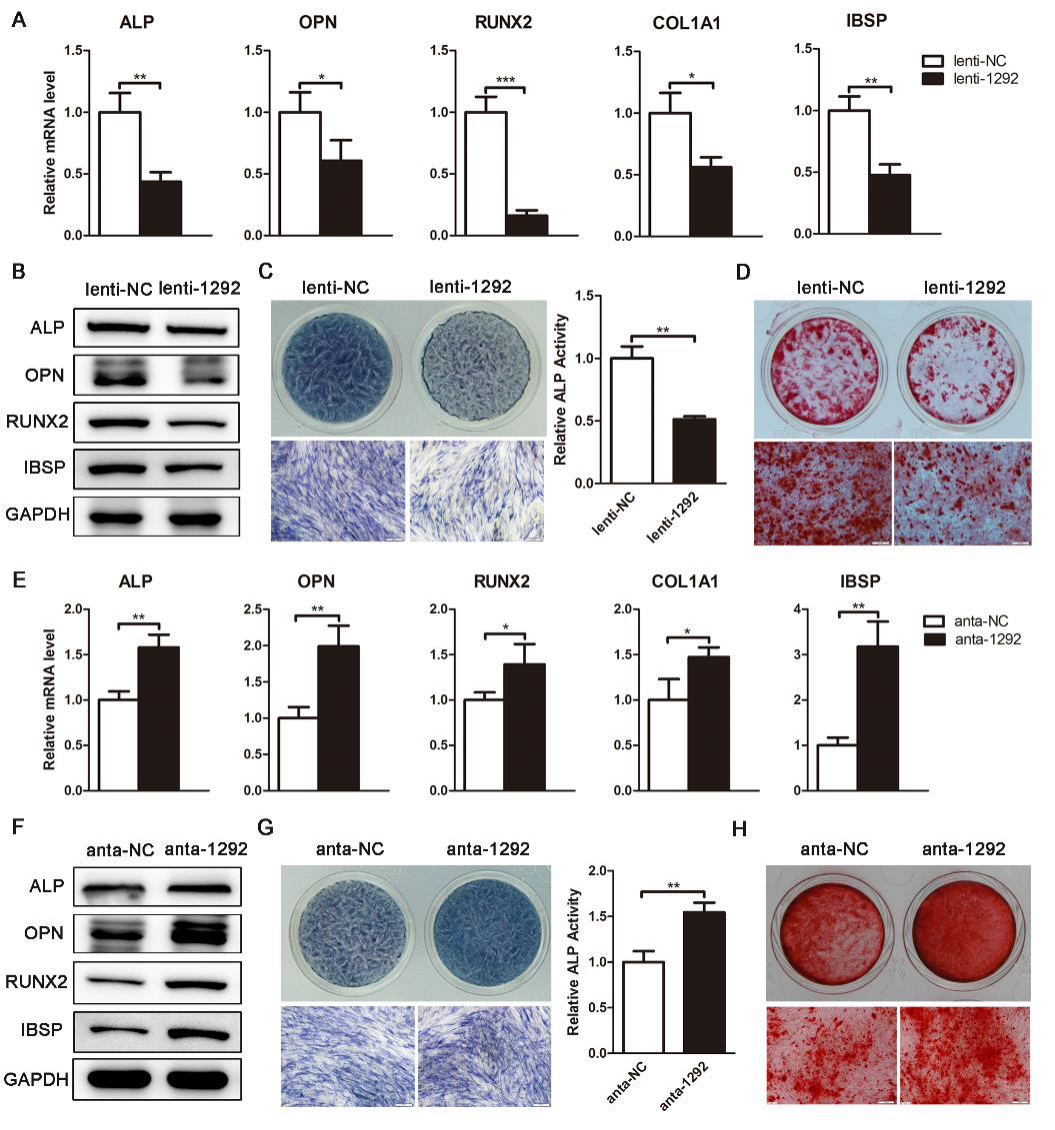
miR-1292 inhibits osteoblast differentiation. (A) The expression of osteoblast-related genes was analyzed by qRT-PCR on day 6 of hADSC osteogenic differentiation. (B) Western blot analysis of protein levels of osteogenic markers on day 6. (C) ALP staining and ALP activity were assessed on day 4 of osteoblast differentiation. (D) Alizarin red staining was performed to indicate mineral deposition on day 15. (E) qRT-PCR was performed to analyze the expression of osteoblast marker genes on day 6. (F) Western blot analysis of the protein levels of osteogenic markers on day 6. (G) ALP staining and ALP activity were assessed on day 4 of osteogenic differentiation. (H) Alizarin red staining was performed to indicate mineral deposition on day 15. GAPDH was used as an internal control for western blot analysis. qRT-PCR data were normalized to GAPDH expression, results are presented as the mean ± SD, n = 3. ^*^P < 0.05, ^**^P < 0.01, ^***^P < 0.001 compared to the control. Scale bars: 200 μm.

### miR-1292 expression increases with cell senescence and declines during the osteogenesis of hADSCs

To identify miRNAs displaying critical regulatory effects on cellular senescence and osteogenic differentiation, we performed a microarray to compare differential expression patterns between MSCs and differentiated osteogenic cells after 4 days. We found a significantly decreased expression of miR-1292 with differentiation of hADSCs into osteoblasts (data not shown). During hADSC senescence, miR-1292 expression was gradually increased, as measured by miRNA-specific stem-loop RT-PCR (Fig. 2A). We also detected the expression level of miR-1292 in adipose tissues from different female donors whose ages were 17, 28, and 68 (F17, F28, and F68, respectively), results showed that miR-1292 was upregulated in cells from the aged individual compared to that in cells from young donors (Supplementary Fig. 1). Additionally, the expression level of miR-1292 was decreased in a time-dependent manner during osteogenic differentiation (Fig. 2A). Sequence analysis revealed that miR-1292 is highly conserved among *Homo sapiens*, *Pan troglodytes*, and *Pongo pygmaeus* (Fig. 2B).

Furthermore, we analyzed the expression pattern of miR-1292 in different mouse tissues and detected high levels in the pancreas, but extremely low levels in the bone and hADSC-derived osteoblasts (M-osteoblasts). Also, miR-1292 exhibited relatively low expression in M-osteoblasts compared to that in other common cell lines (Fig. 2C). Moreover, we analyzed correlations between the expression of miR-1292 and senescence-associated factors in 70 clinical bone specimens. Results showed that the expression of miR-1292 was positively correlated with P16 and P21 levels (Fig. 2D; Supplementary Fig. 2A). We also measured correlations between the expression of bone formation markers and miR-1292 in bone samples. ALP, OPN, and RUNX2 were found to be positively correlated with each other and negatively correlated with the expression of miR-1292 (Fig. 2D; Supplementary Fig. 2B-D). Moreover, negative correlations were detected between senescent markers and bone formation factors in these bone samples (Supplementary Fig. 2E-J). These findings suggested that miR-1292 might promote senescence and repress osteogenic differentiation in hADSCs.

**Figure 5.**
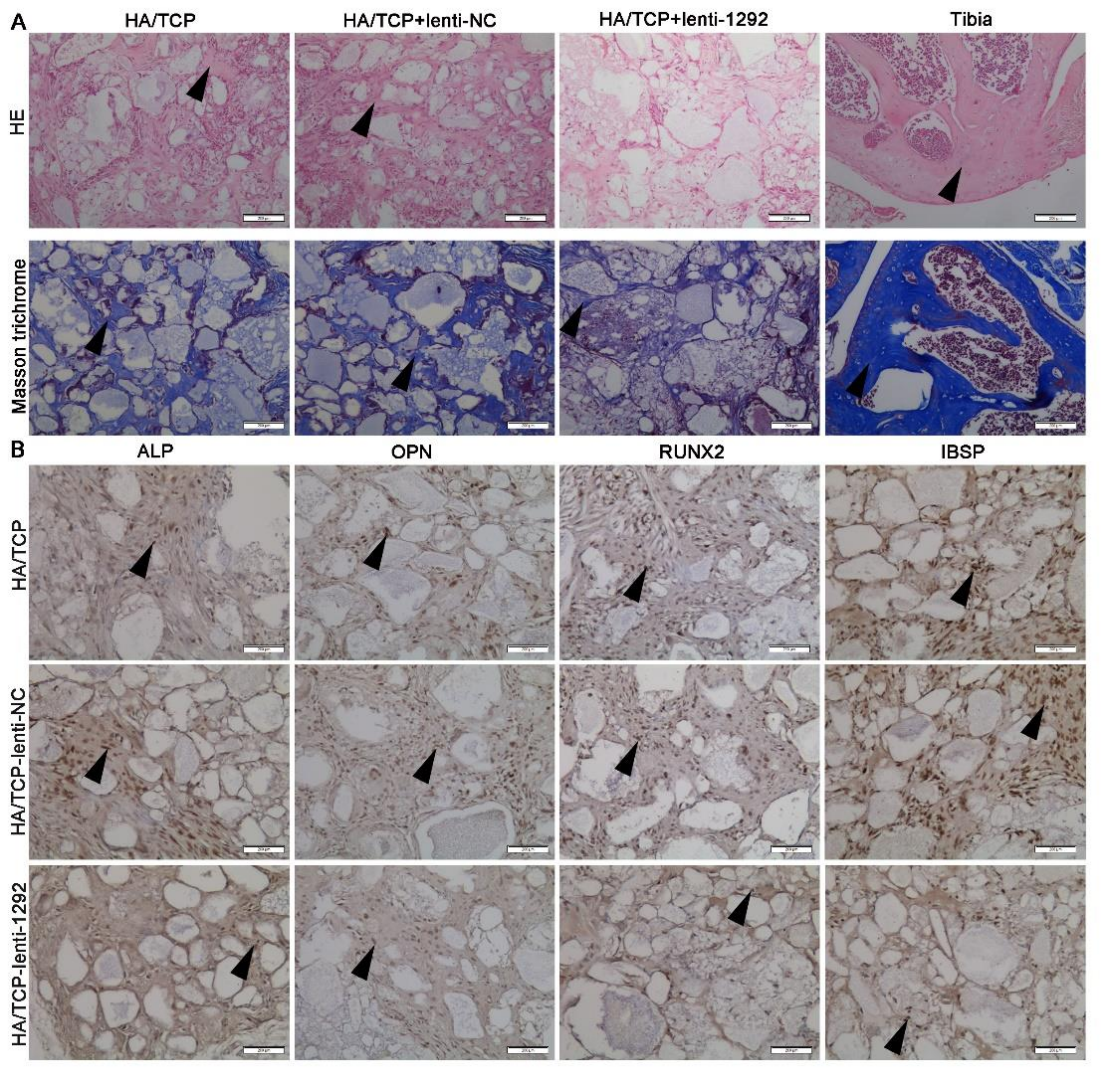
miR-1292 impairs ectopic bone formation of hADSCs *in vivo*. (A) H&E staining indicated osteoid formation and Masson trichrome staining was performed to analyze collagen deposition in xenografts. (B) Immunohistochemical staining showing the expression of osteogenic markers in xenografts. Black arrows represent positive signals. Scale bars: 200 μm.

**Figure 6.**
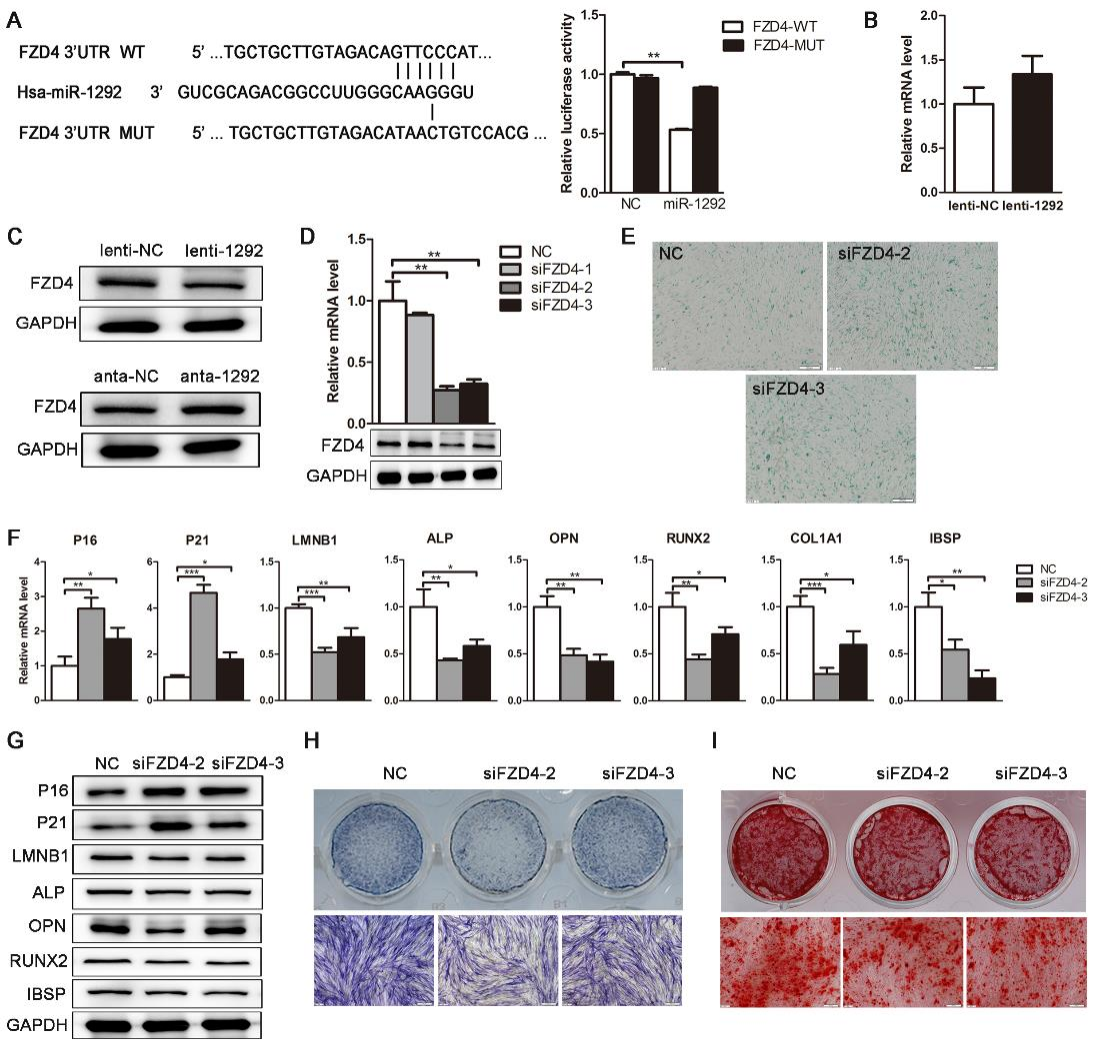
FZD4 is a direct target of miR-1292. (A) Bioinformatics analysis was performed to predict the miR-1292-binding seed sequence in the 3′ UTR of *FZD4*. Wild-type (WT) or mutant-type (MUT) constructs were inserted into the psiCHECK-2 reporter vector. Dual luciferase activity was measured in the lysates of different groups. (B and C) qRT-PCR and western blot were performed to analyze FZD4 expression in hADSCs. (D) The knockdown efficiency of three FZD4-specific siRNAs was confirmed by qRT-PCR and western blot. (E) SA-β-gal staining was performed after FZD4 knockdown. (F and G) The expression of critical regulators of cellular senescence and osteogenesis was analyzed by qRT-PCR and western blot. (H) ALP staining was performed on day 4 after FZD4 knockdown. (I) Mineral deposition was detected by alizarin red staining on day 15 of osteogenic differentiation. GAPDH was used as an internal control for western blot analysis and qRT-PCR data were normalized to PPIA or GAPDH expression; results are presented as the mean ± SD, n = 3. ^*^P < 0.05, ^**^P < 0.01, ^***^P<0.001 compared to the control. Scale bars: 200 μm.

### miR-1292 regulates the cellular senescence of hADSCs

To investigate the functional effect of miR-1292 on hADSC senescence, we overexpressed miR-1292 by expressing its precursor in hADSCs using a lentiviral vector (lenti-1292). Mature miR-1292 levels were elevated by approximately 300-fold compared to those in lenti-NC-treated cells 48 h after infection as confirmed by miRNA specific stem-loop RT-PCR (Fig. 3A). qRT-PCR and western blot analyses also showed that lenti-1292-infected hADSCs expressed higher levels of the senescence markers P16 and P21 and lower expression of LMNB1 than control cells (Fig. 3B, C). Moreover, the number of SA-β-gal-stained cells was increased after miR-1292 overexpression (Fig. 3D). Further, we used a specific antagomir (anta) to reduce endogenous expression of miR-1292. qRT-PCR analysis showed that transfection of anta-1292 effectively inhibited miR-1292 expression in hADSCs (Fig. 3E). Anta-1292 transfection also resulted in the significant downregulation of cell senescence markers at the mRNA and protein levels, in addition to reduced SA-β-gal secretion (Fig. 3F-H), indicating that suppression of miR-1292 can inhibit senescence in hADSCs. Hence, these data confirmed that miR-1292 is a positive regulator of hADSC senescence.

### miR-1292 suppresses the osteogenic differentiation of hADSCs in vitro

To further elucidate the effect of miR-1292 on osteogenic differentiation, lentivirus-infected hADSCs were induced to differentiate into the osteogenic lineage. As determined by qRT-PCR, mRNA levels of osteoblast-specific genes, including *ALP*, *OPN*, *RUNX2*, *COL1A1*, and *IBSP* were significantly downregulated on day 6 of osteogenesis with miR-1292 overexpression (Fig. 4A). Meanwhile, the expression of osteoblast markers at the protein level was attenuated (Fig. 4B). ALP staining and ALP activity assays showed that the expression and activity of ALP were decreased in lenti-1292-infected cells (Fig. 4C). Alizarin red staining indicated that matrix mineralization was also reduced in lenti-1292-infected cells (Fig. 4D). These data demonstrated that overexpression of miR-1292 inhibits the osteogenic differentiation of hADSCs *in vitro*. To further confirm the effect of miR-1292 inhibition on osteogenic differentiation, anta-1292- or anta-NC-transfected hADSCs were induced to differentiate into the osteogenic lineage. The downregulation of miR-1292 significantly enhanced osteoblast differentiation, which was indicated by the upregulation of osteoblast-specific markers at the mRNA and protein level, increased ALP expression and ALP activity, and enhanced matrix mineralization in anta-1292-transfected hADSCs compared to those in cells transfected with anta-NC (Fig. 4E-H). Collectively, our results implied that miR-1292 is a negative regulator of hADSC osteoblast differentiation.

### miR-1292 impairs bone formation in vivo

For the functional evaluation of miR-1292 *in vivo*, we established a preclinical ectopic bone formation model in NOD/SCID mice according to a previous study [34]. HA/TCP, HA/TCP loaded with lenti-NC or HA/TCP loaded with lenti-1292 was implanted subcutaneously into NOD/SCID mice. After 8 weeks, H&E staining revealed a dramatical decrease in osteoid formation in the mouse grafts with lenti-1292-infected cells, whereas enhanced osteoid generation was detected in the grafts with either lenti-NC-infected cells or HA/TCP group (Fig. 5A). Masson trichrome staining revealed a reduced production of collagen by miR-1292-overexpressing cells in comparison to the lenti-NC group (Fig. 5A). Besides, immunohistochemistry staining showed that the expression of ALP, OPN, RUNX2, and IBSP was decreased in mouse grafts with lenti-1292-infected cells (Fig. 5B). These results demonstrated that overexpression of miR-1292 inhibits ectopic bone formation *in vivo*.

### miR-1292 regulates hADSC senescence and osteogenesis by directly targeting FZD4

To elucidate the molecular mechanisms that underlie miR-1292-mediated hADSC senescence and osteogenic regulation, we searched for potential targets of miR-1292 using the miRNA target prediction databases TargetScan, PicTar and miRanda. Among hundreds of predicted targets, we identified that senescence-associated and osteogenic genes *NFAT5*, *GDNF*, *FZD4*, *FZD5,* and *CLCN5* have miR-1292 binding sites in their 3′ UTRs (Fig. 6A; Supplementary Fig. 3A). To investigate whether miR-1292 directly target these genes, we inserted the wild-type (WT) or mutant (MUT) 3′ UTR sequences of these genes, containing the miR-1292-binding site, into the dual luciferase construct psi-CHECK2. 293T cells were co-transfected with the luciferase vector and miR-1292 agomir or miR-NC. Luciferase activity was then measured in triplicate 48 h after transfection. Results showed that miR-1292 overexpression remarkably inhibited luciferase reporter activity from the vector containing the WT FZD4 3′ UTR, but not from the mutated FZD4 or those of other genes (Fig. 6B; Supplementary Fig. 3B). Additionally, after miR-1292 overexpression, FZD4 was markedly downregulated at the protein level, but not at mRNA level (Fig. 6C, D), suggesting that miR-1292 regulates FZD4 expression at the post-transcriptional level in hADSCs. Also, qRT-PCR results showed that the expression of miR-1292 was negatively correlated with FZD4 in 70 clinical bone samples (Supplementary Fig. 4A). We also identified negative correlations between the expression of FZD4 and senescent factors while significantly positive associations existed between FZD4 and bone formation markers (Supplementary Fig. 4B-F). To determine the role of FZD4 in hADSC senescence and osteogenic differentiation, we suppressed FZD4 expression with siRNAs. qRT-PCR and western blot analyses confirmed that siFZD4-2 and siFZD4-3 effectively inhibited FZD4 expression at both the mRNA and protein levels (Fig. 6D). Moreover, the number of SA-β-gal-stained senescent cells was markedly increased upon FZD4 downregulation (Fig. 6E). qRT-PCR and western blot analyses showed that the mRNA and protein levels of senescence-associated markers P16 and P21 were increased, whereas LMNB1 and osteogenic factors were sharply decreased in siFZD4 transfected hADSCs compared to expression in siNC-transfected cells (Fig. 6F, G). Besides, decreases in ALP and alizarin red staining indicated that the suppression of FZD4 inhibits the osteogenic differentiation of hADSCs (Fig. 6H, I). These results were consistent with those observed in miR-1292-overexpressing hADSCs, suggesting that miR-1292 functionally targets FZD4 to regulate hADSC senescence and osteogenic differentiation.

### Knockdown of FZD4 can remedy the effect of endogenous miR-1292 reduction on senescence and osteogenesis

**Figure 7.**
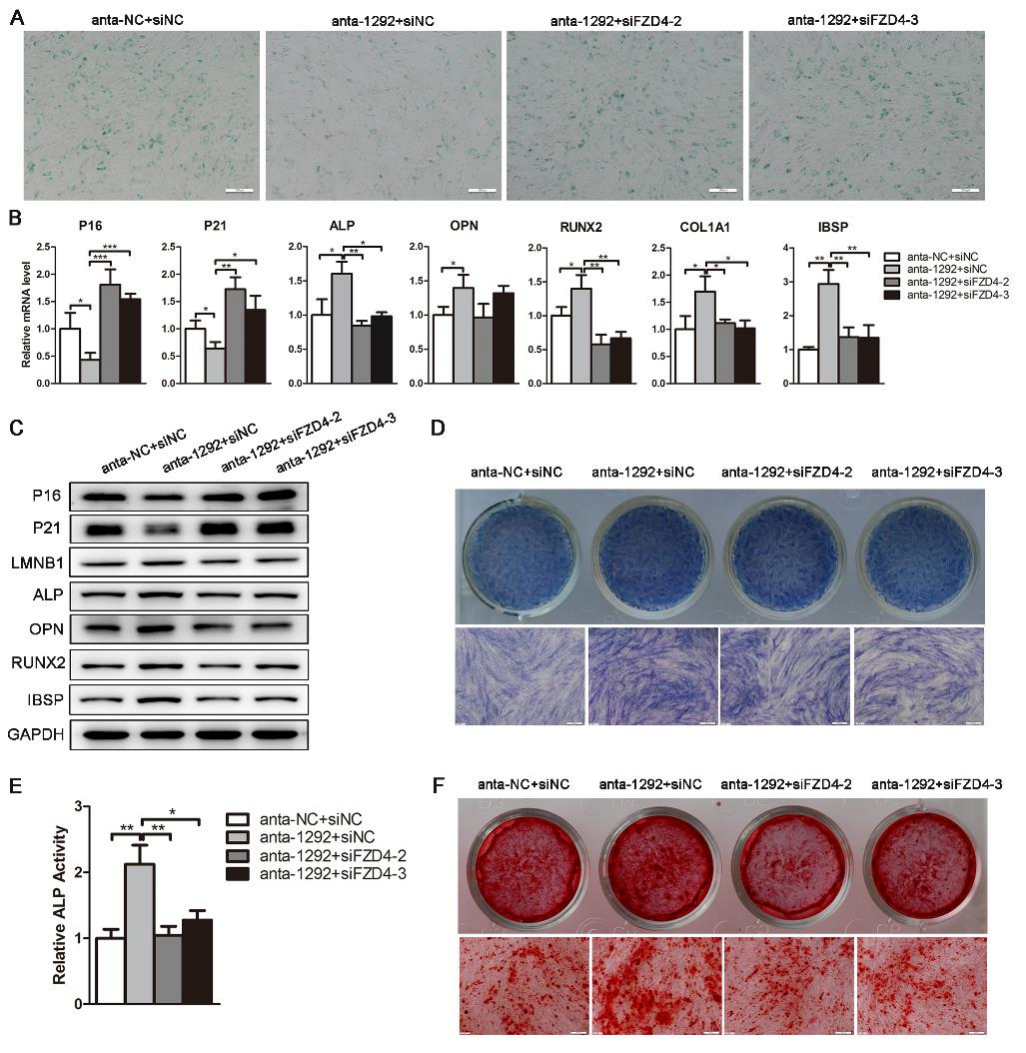
FZD4 knockdown reverses the effect of miR-1292 inhibition on cellular senescence and osteogenic differentiation of hADSCs. (A) SA-β-gal staining was performed to detect SA-β-gal secretion. (B) qRT-PCR was used to analyze senescence-associated and osteogenic markers expression after different treatments. (C) Western blot was performed to analyze the expression of cellular senescence and osteogenic factors. (D and E) ALP staining and ALP activity assays were performed on day 4 of osteogenic differentiation. (F) Alizarin red staining was performed to indicate calcification on day 12 of osteogenic differentiation. GAPDH was used as an internal control for western blot analysis, whereas qRT-PCR data were normalized to PPIA or GAPDH; results are presented as the mean ± SD, n = 3. ^*^P < 0.05, ^**^P < 0.01, ^***^P < 0.001 compared to the control. Scale bars: 200 μm.

To further confirm that FZD4 mediates the effect of miR-1292 on hADSC senescence and osteogenic differentiation, we suppressed miR-1292 and then silenced FZD4 expression in hADSCs. We found inhibition of cellular senescence and promotion of osteogenesis in cells transfected with anta-1292, as compared to those in the anta-NC group. However, FZD4 knockdown effectively abolished the suppression of anta-1292 on hADSC senescence, as demonstrated by increased SA-β-gal staining and elevated expression of senescence-associated markers at the mRNA and protein levels (Fig. 7A-C). Also, FZD4 knockdown effectively reversed the promoting effect of anta-1292 on hADSC osteogenic differentiation, as indicated by downregulation of osteogenic markers at the mRNA and protein levels, decreased ALP staining and ALP activity, and reduced mineral deposition (Fig. 7B-F). These results demonstrated that deletion of its target could block the effect of anta-1292, further implying that miR-1292 regulates the senescence and osteogenic differentiation of hADSCs by functionally targeting and repressing FZD4.

### miR-1292 regulates senescence and osteoblast differentiation in hADSCs via the Wnt/β-catenin signaling pathway

**Figure 8.**
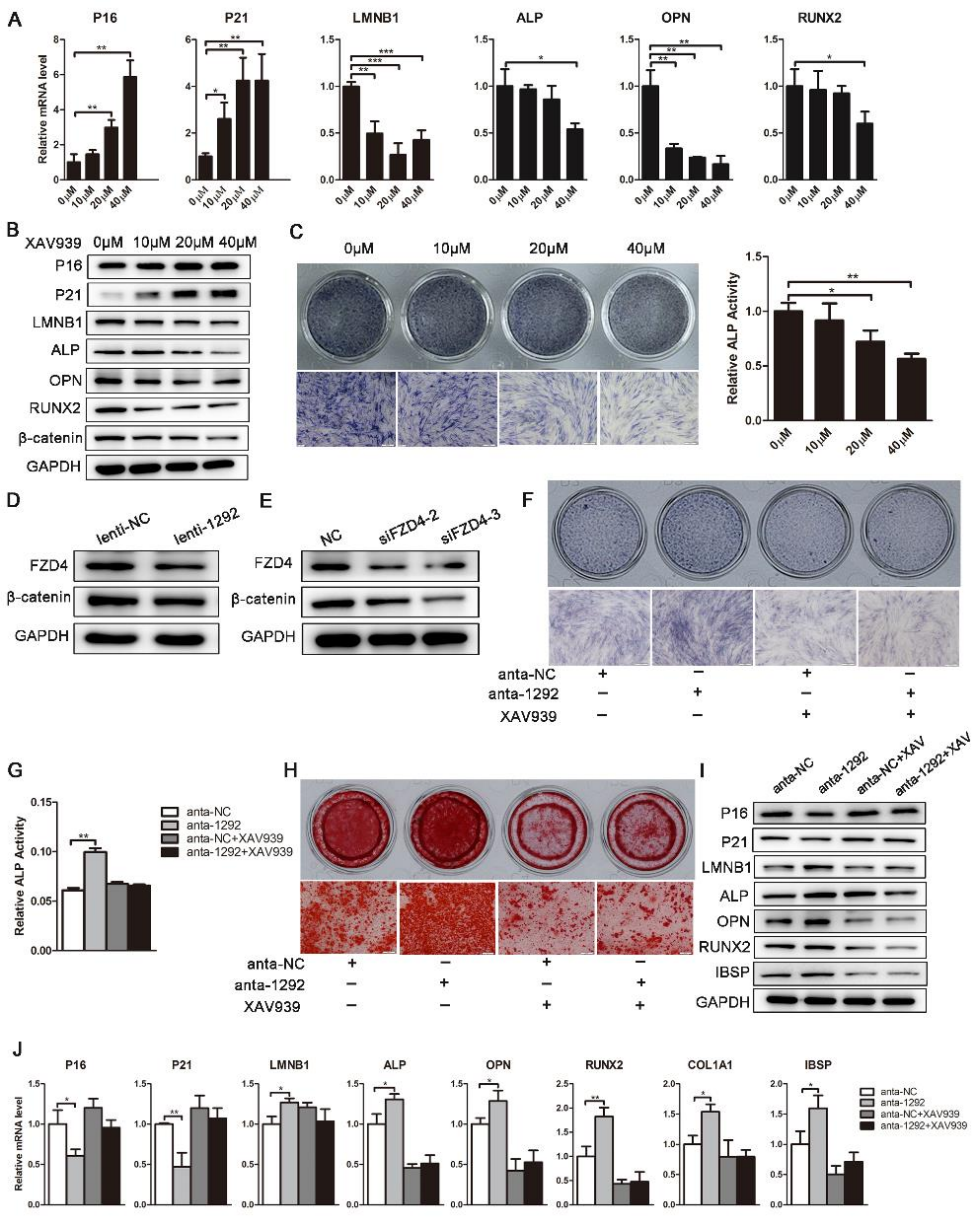
miR-1292 regulates hADSC senescence and osteogenic differentiation through Wnt/β-catenin signaling. (A) The Wnt/β-catenin inhibitor XAV939 was used to block this pathway. The expression of senescence-associated markers and osteogenesis-related genes was analyzed by qRT-PCR after different treatments. (B) Western blot was performed to analyze the protein levels of senescence markers, osteogenic factors, and β-catenin after treatment with XAV939. (C) ALP staining and activity assays were used to analyze the effects of different concentrations of XAV939 on osteogenesis. (D and E) Western blot was performed to analyze the protein levels of FZD4 and β-catenin after miR-1292 overexpression or FZD4 downregulation in hADSCs. (F and G) ALP staining and ALP activity were measured on day 4 of osteogenic differentiation. (H) Alizarin red staining was performed to indicate calcification. (I) The protein levels of senescence-associated and osteogenic factors were detected by western blot. (J) qRT-PCR was performed to analyze the mRNA levels of senescence- and osteogenesis-related genes after different treatments. GAPDH was used as an internal control for western blot. qRT-PCR data were normalized to PPIA or GAPDH expression; results are presented as the mean ± SD, n = 3. ^*^P < 0.05, ^**^P < 0.01, ^***^P < 0.001 compared to the control. Scale bars: 200 μm.

**Figure 9.**
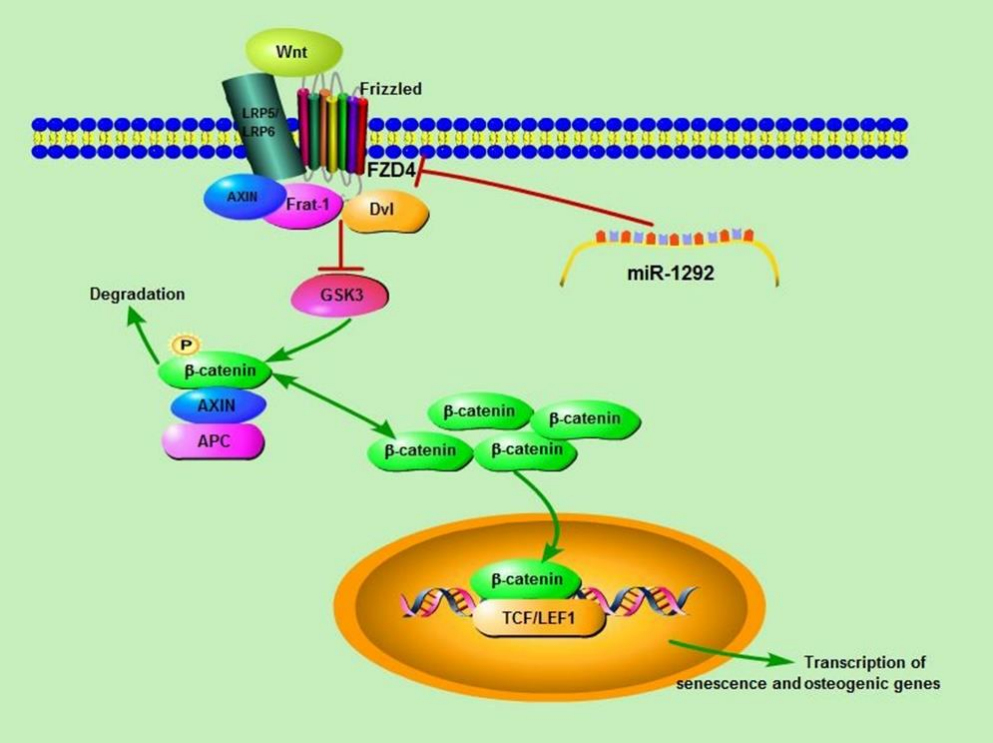
Schematic diagram of the regulatory effects of miR-1292 on hADSC senescence and osteoblastic differentiation. The Wnt protein binds to the receptor FZD4 on the cell membrane and inhibits GSK3 activity through a mechanism involving Axin, Frat-1, and Disheveled (Dvl). Intracellular β-catenin accumulates and translocates to the nucleus, where it binds TCF/LEF1 and induces the transcription of senescence and osteogenesis-associated genes. After ectopic expression of miR-1292, the FZD4 expression is significantly inhibited, leading to decreased β-catenin expression, enhanced cellular senescence, and reduced osteogenic differentiation.

FZD4, a member of the frizzled gene family, is coupled with the Wnt/β-catenin canonical signaling pathway. To further determine if miR-1292 promotes hADSC senescence and inhibits osteogenesis through the Wnt/β-catenin pathway, the specific inhibitor XAV939 was added to the cell culture or osteogenic induction medium. We found that XAV939 remarkably enhanced hADSC senescence and suppressed osteogenic differentiation of hADSCs in a concentration-dependent manner, which was confirmed by the elevated expression of P16 and P21 at the mRNA and protein levels, decreased expression of LMNB1 and osteogenic factors, and reduced ALP staining and ALP activity (Fig. 8A-C). Moreover, β-catenin was strikingly downregulated at the protein level when 40 μM XAV939 was added to the medium (Fig. 8B). Western blotting studies revealed that miR-1292 overexpression decreased both FZD4 and β-catenin expression, indicating that Wnt/β-catenin signaling was impaired (Fig. 8D). This finding was consistent with the effect of FZD4 downregulation on the protein expression of β-catenin (Fig. 8E). Furthermore, enhanced hADSC osteogenesis mediated by miR-1292 downregulation was almost entirely attenuated by XAV939, as indicated by reduced ALP staining, ALP activity, and mineral deposition, as well as decreased expression of osteogenic markers (Fig. 8F-J). Moreover, the inhibition of hADSC senescence by miR-1292 repression was also blocked by XAV939, as demonstrated by the upregulation of P16 and P21 at mRNA and protein levels and the increased expression of LMNB1 (Fig. 8I, J). Thus, miR-1292 regulates hADSC senescence and osteogenic differentiation via the Wnt/β-catenin pathway (Fig. 9).

## DISCUSSION

**Figure 10.**
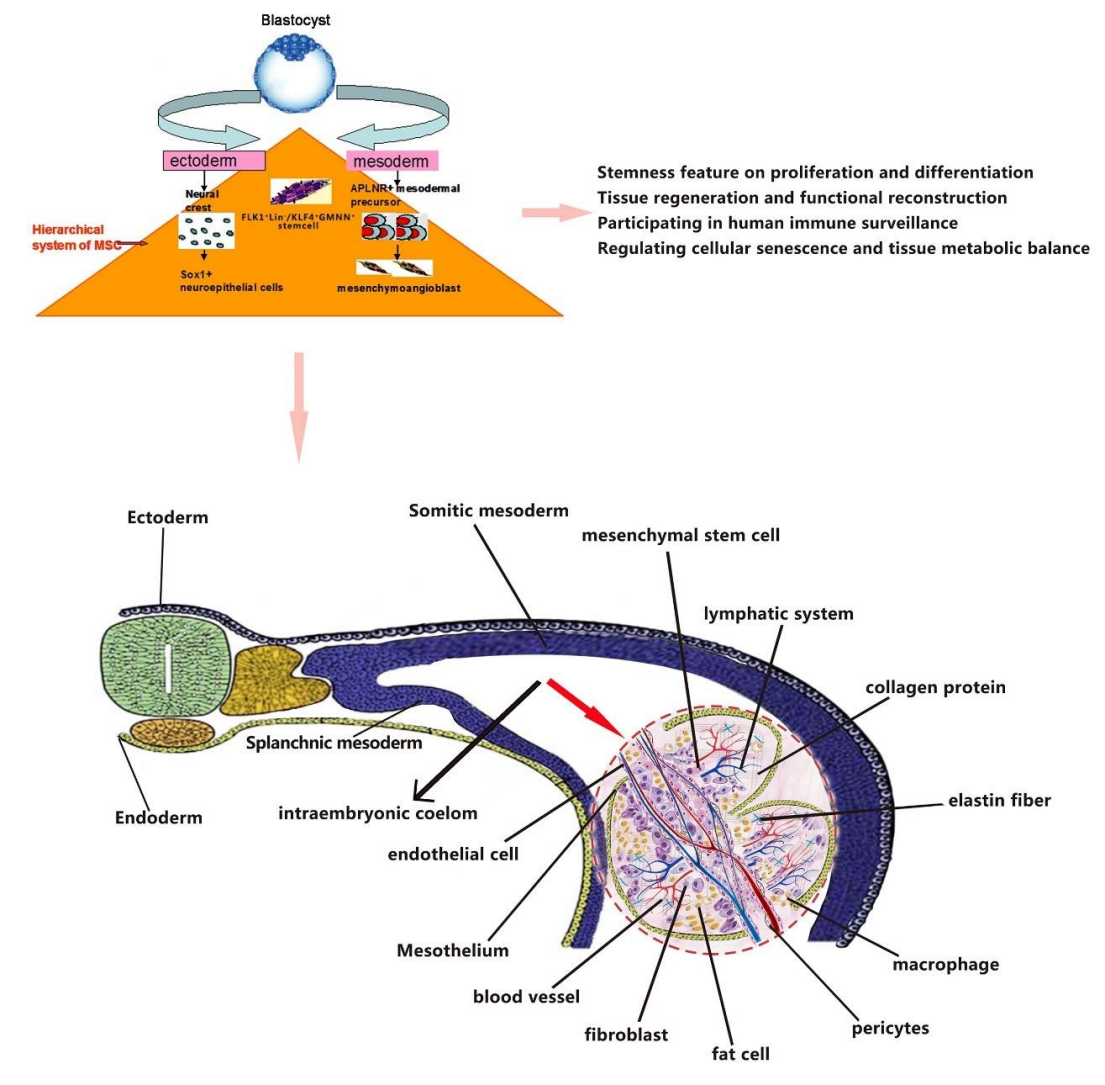
Embryonic development and organization structure of TE/SJ/ mesenchymal tissue system. Mesenchymal tissue system consists of mesodermal-derived all kinds of cells (Flk^+^Lin^-^/KLF4^+^GMNN^+^) from postembryonic subtotipotent stem cells to functional cells in different stages of the embryonic development. In mammals, the intraembryonic coelom is a fluid-filled cavity with the somatic and the visceral mesoderm layers, which then forms TE/SJ/mesenchymal tissue system. Meanwhile, TE/SJ/mesenchymal tissue system constitutes the source of major organs and is the connecting pathway between them. It is involved in the proliferation and differentiation of stem cells, tissue regeneration, and functional reconstruction. It also participates in human immune surveillance, cellular senescence and tissue metabolic.

The development of science and technology has brought revolutionary progress towards understanding tissue and organ function. It is gradually emerging that some scientific discoveries in medical science are compatible with the principles of ancient medicine. In traditional Chinese medicine (TEM), there is a theory of Zang-Fu organs characterized five Zang (Yin) organs which comprise solid organs such as the Heart, Spleen, Lung, Kidney, and Liver. Each of these organs is paired respectively with a hollow or Fu organ, the Small Intestine, Stomach, Large Intestine, Urinary Bladder, and Gall Bladder, implying that the sixth organ of Fu, TE/SJ is visible, however, not anatomically found. We consider TE/SJ is a real space originated from intraembryonic coelom with the somatic and the visceral mesoderm layers, which later forms TE/SJ/mesenchymal tissue system. The zygote is the first stem cell of the human body with all innate Qi. The innate Qi drives the zygote to differentiate into tissues and organs. Stem cells are the carrier of Qi, and Qi is the driving force for stem cell proliferation and differentiation, they are interdependent and inseparable. The embryo grows in mother’s body and continues developing after birth, whereas the acquired Qi is produced by nutrient and oxygen in TE/SJ/stem cell system guided by the innate Qi. On the other hand, TE/SJ originates from the MSC system during embryonic development. The MSC system consists of all mesoderm-derived functional cells at different stages of embryonic development, including postembryonic subtotipotent stem cells and progenitor cells covering all subsets of MSCs, such as CXCL12-abundant reticular (CAR) cells and pericytes [35]. Recently, the group from Neil used a probe-based confocal laser endomicroscopy (pCLE) during endoscopy and observed a submucosal “reticular pattern” consisting of dark branching bands surrounding large, fluorescein-filled polygonal spaces in the extrahepatic bile ducts and pancreatic ducts [36]. We demonstrated for the first time the cellular structure and functional essence of TE/SJ. Our study reflects not only the investigation of molecular mechanisms but also the analysis of mesenchymal tissue system from the perspective of the human organ system. The results suggest that TE/SJ/mesenchymal tissue system is a vital organ to maintain the metabolism and regulate cell senescence of human organs and tissues. We propose that TE/SJ as well as MSC system forms a network of functional cavities between the organs and could regulate endocrine system metabolism, immune balance, tissue regeneration and functional reconstruction (Fig. 10).

The imbalance of Qi is the fundamental mechanism of aging and the principal reason for some age-related diseases. Qi decreases along with stem cells while the function of TE/SJ/mesenchymal tissue system deteriorates in the process of aging, illness, and death. Therefore, treatment strategies aimed at enhancing the differentiation capacity and function of stem cells in TE/SJ/mesenchymal tissue system need to understand that increasing Qi is of significance for anti-aging. MSCs have attracted considerable attention in the fields of tissue engineering and regenerative medicine due to the aforementioned intrinsic capacities. However, serial passages of MSCs in culture lead to loss of differentiation potential and stem cell characteristics, which eventually results in the induction of cellular senescence [37]. Genetic and epigenetic mechanisms tightly regulate all of these processes. miRNAs are crucial regulators of cellular senescence and are involved in the fate determination of MSCs. In this study, we provided evidence for the first time that miR-1292 enhances senescence and attenuates osteoblast differentiation of hADSCs *in vitro*, as well as suppresses bone formation *in vivo*. In clinical bone samples, miR-1292 was positively correlated with the expression of senescence-associated factors and negatively associated with the expression of bone formation markers. FZD4, an essential receptor of the Wnt/β-catenin signaling pathway, was identified as a direct target of miR-1292. Our findings suggested that miR-1292 might be an effective agent in bone tissue engineering, bone repair, and fracture healing.

Osteoporosis, characterized by decreased bone mass and microarchitectural deterioration of bone tissue, increases the propensity of bone fracture. Primary osteoporosis comprises bone loss attributed to aging or a decline in sex hormones associated with aging [38,39]. A decrease in the number and osteogenic capacity of MSCs is one of the leading causes of age-related bone loss [40-42]. Some studies have indicated that the osteogenic activity of MSCs deteriorates progressively as a function of increasing lifespan [43]. In our study, we established a replicative MSCs senescence model to study the mechanisms of aging. Expansion of MSCs in culture results in their accelerated aging, displaying enlarged morphology, decreased expression of MSC-specific surface antigens, and increased activity of lysosomal acid β-galactosidase [44]. The differentiation potential during MSC senescence is controversial, but most of the studies have shown a decrease in their osteogenic differentiation capacity. In one well-controlled study, extremely long-term passaging of MSCs resulted in diminished *in vitro* osteogenic differentiation potential [45]. Mueller et al. used a three dimensional (3D) culture system and qRT-PCR methods to test the osteogenic potential of human bone marrow stem cells (hBMSCs) and found that the osteogenic potential of BMSCs decreases with age [46]. A study by Vacanti et al. revealed that a significant decrease in the differentiation potential of cultured porcine BMSCs into osteogenic lineage in the late passages as compared to early passages [47]. Moreover, while aged MSCs maintain a particular ability to differentiate toward the adipogenic lineage regardless of the expansion condition, their osteogenic potential is significantly impaired by senescence [48]. Here, we showed that replicative senescence reduces the osteogenic potential of MSCs. Thus, treatment strategies aimed at enhancing MSC osteoblast differentiation ability and/or preventing the senescence of these cells could lead to bone growth and combat osteoporosis.

As important regulatory factors, miRNAs are key regulators and reliable markers of cellular senescence and play crucial roles in stem cell proliferation and differentiation [49]. miRNAs control the senescence of stem cells by targeting genes involved in DNA damage, epigenetic changes, and metabolism [50]. The expression of miR-335 has been reported to be increased in hBMSCs from old donors and in senescent MSCs induced by diverse stimuli [25]. miR-543 and miR-590-3p also have been reported to regulate the cellular senescence of human MSCs via the suppression of the AIMP3/p18 pathway [51]. Furthermore, a growing body of evidence has shown that miRNAs are critical for the regulation of osteogenic differentiation. miR-216a was found to be dramatically increased during osteogenic differentiation and markedly promoted osteogenic differentiation of hADSCs [52], whereas miR-10b enhanced osteogenic differentiation and inhibited adipogenic differentiation of hADSCs [53]. Furthermore, increased expression of miR-486-5p has been observed in response to the replicative senescence, and overexpression of miR-486-5p induced premature senescence as well as inhibited osteogenic differentiation of hADSCs [54]. These findings suggest that miRNAs play essential roles in regulating MSC senescence and osteogenesis. We, therefore, analyzed miRNA expression profiles during hADSC osteogenic differentiation and found a dramatic downregulation of miR-1292 during osteogenesis. Moreover, miR-1292 was positively correlated with the expression of senescence-associated factors and negatively associated with bone formation markers in clinical bone samples. A series of functional assays in our study further confirmed that miR-1292 could enhance hADSC senescence, impair osteogenesis *in vitro*, and notably suppress bone tissue generation *in vivo*. Our findings suggested that miR-1292 might represent a potential therapeutic target and biomarker for treating age-related bone metabolic disorders.

Most miRNAs post-transcriptionally regulate gene expression by inducing mRNA degradation and/or repressing mRNA translation through binding to complementary sequences in the 3′ UTR of target mRNAs [55,56]. Based on bioinformatic analyses, we predicted that the senescence- and osteogenesis-related genes *NFAT5*, *GDNF*, *FZD4*, *FZD5,* and *CLCN5* might be potential targets of miR-1292. Among those genes, frizzled class receptor 4 (FZD4), a vital receptor in Wnt/β-catenin pathway, was further identified as the bona fide target of miR-1292 during the regulation of MSC senescence and osteogenesis. FZD4 is a member of the frizzled gene family, which encodes seven-transmembrane domain proteins that are receptors for the Wingless type MMTV integration site (Wnt) family of signaling proteins. Screening for age-related changes in gene expression in the intima/media of human mammary arteries revealed a modified expression of FZD4 with age [57]. Besides, FZD4 has been demonstrated to regulate hBMSC osteogenesis [58]. miRNA-199a-5p can also remarkably increase the inhibitory effect of dexamethasone on osteoblast proliferation by inhibiting FZD4 and WNT2 expression [59]. As a critical factor involved in Wnt/β-catenin signaling, FZD4 has also been reported to participate in multiple processes mediated by miRNAs. For example, miR-199a-5p can regulate myogenesis through the suppression of FZD4 [60]. miR-377 also controls the proliferation, apoptosis, migration, and invasion in metastatic prostate cancer cells by targeting FZD4 [61] whereas miR-101 suppresses the WNT5a-induced proliferation of lung fibroblasts by targeting FZD4/6 [62]. In our study, we found that the downregulation of FZD4 can enhance senescence and suppress osteoblast differentiation of hADSCs, and that knockdown of FZD4 can rescue the effect of endogenous miR-1292 suppression on cellular senescence and osteogenesis. Our findings indicate that miR-1292 plays a vital role in modulating stem cell senescence and osteogenic differentiation by directly targeting FZD4.

Wnts comprise a family of 19 secreted glycoproteins that mediate a large number of cellular and biological processes such as embryogenesis, organogenesis, and tumorigenesis [63,64]. Canonical WNT signals are transduced through Frizzled (FZD) family receptors and LRP5/LRP6 co-receptors to induce the β-catenin signaling cascade, and Norrin/FZD4 signaling activates the canonical Wnt pathway [65-68]. By mediating the expression of different target genes, Wnt/β-catenin has a bidirectional regulatory role and has both enhancing and suppressive effects on cellular senescence [69-71]. Canonical Wnt/β-catenin signaling also supports osteoblastogenesis through many mechanisms including stem cell renewal, the induction of preosteoblast replication, the stimulation of osteogenesis, and the inhibition of osteoblast and osteocyte apoptosis [14,72]. The Wnt-dependent nuclear accumulation of β-catenin is a significant trigger of osteoblastic differentiation, and bone formation and its endogenous inhibitors Dkk-1 and sclerostin comprise potential therapeutic targets to enhance osteoblastic bone formation [73,74]. We demonstrated that the Wnt/β-catenin antagonist XAV939, which selectively inhibits β-catenin-mediated transcription, promotes cell senescence and inhibits osteogenic differentiation in a dose-dependent manner. XAV939 almost wholly blocked the augmentation of senescence and inhibition of osteogenic differentiation in hADSCs induced by miR-1292. These findings revealed that miR-1292 accelerates senescence and represses osteogenic differentiation of hADSCs through the Wnt/β-catenin signaling pathway.

In summary, we propose that TE/SJ/mesenchymal tissue system is the largest organ consisting of all functional cells derived from the mesoderm during embryonic development and is responsible for maintaining homeostasis and regulating cell senescence. In TE/SJ/mesenchymal tissue system, miR-1292 is involved in the regulation of cellular senescence, osteoblast differentiation, and the maintenance of bone mass. miR-1292 was found to be positively correlated with senescence and negatively associated with bone formation in clinical bone samples; further, it aggravated MSC senescence, inhibited osteoblast differentiation *in vitro*, and suppressed ectopic bone formation *in vivo*. Mechanistically, miR-1292 was found to regulate the senescence and osteogenic differentiation of hADSCs by directly targeting FZD4 and suppressing Wnt/β-catenin signaling. These findings expand our knowledge of TE/SJ and MSC system, and the miR-1292/FZD4/Wnt regulatory circuit could facilitate the development of novel targeted therapeutics to treat patients who have osteoporosis and other age-related skeletal diseases.

This work was supported by grants from the National Natural Science Foundation of China (No. 81700782, 81370466, 81370879, 81672313), the National Key Research and Development Program of China (2016YFA0101000, 2016YFA0101003), and CAMS Innovation Fund for Medical Sciences (2017-I2M-3-007).

## Supplementary Materials

The Supplemenantry data can be found online at: www.aginganddisease.org/EN/10.14336/AD.2018.1110
