# Supplementary material for: MiR-1292 Targets FZD4 to Regulate Senescence and Osteogenic Differentiation of Stem Cells in TE/SJ/Mesenchymal Tissue System via the Wnt/β-catenin Pathway
Source: Aging Dis. 2018 Dec 4;9(6):1103–21. doi: 10.14336/AD.2018.1110 (PMC6284756; doi:10.14336/AD.2018.1110)
Supplement: Supplementary file 1 — The Supplemenantry data can be found online at: www.aginganddisease.org/EN/10.14336/AD.2018.1110 [file AD-9-6-1103-s.pdf]

SUPPLEMENTARY DATA

MiR-1292 Targets FZD4 to Regulate Senescence and Osteogenic Differentiation of Stem Cells in TE/SJ/Mesenchymal Tissue System via the Wnt/β-catenin Pathway

Junfen Fan[1,#], Xingyan An[1,#], Yanlei Yang[1], Haoying Xu[1], Linyuan Fan[1], Luchan Deng[1], Tao Li[2,§], Xisheng Weng[2], Jianmin Zhang[3], Robert Chunhua Zhao[1,*]

[1]Institute of Basic Medical Sciences Chinese Academy of Medical Sciences, School of Basic Medicine Peking Union Medical College, Center of Excellence in Tissue Engineering Chinese Academy of Medical Sciences, Beijing Key Laboratory (No. BZO381), Beijing, China.
[2]Department of Orthopaedic Surgery, Peking Union Medical College Hospital, Peking Union Medical College, Beijing, China.
[3]Department of Immunology, Research Center on Pediatric Development and Diseases, Institute of Basic Medical Sciences Chinese Academy of Medical Sciences and School of Basic Medicine Peking Union Medical College, State Key Laboratory of Medical Molecular Biology, Beijing, China

[§]Current address: Department of Orthopaedic Surgery, The Affiliated Hospital of Qingdao University, Qingdao, China.

© 2018. Fan J et al. Published online at http://www.aginganddisease.org/EN/10.14336/AD.2018.1110

SUPPLEMENTARY DATA

Supplementary Table 1. The primers of related genes.

Gene name	Forward sequence (5'-3')	Reverse sequence (5'-3')
P16	GATCCAGGTGGGTAGAAGGTC	CCCCTGCAAACTTCGTCCT
P21	TGTCCGTCAGAACCCATGC	AAAGTCGAAGTTCCATCGCTC
LMNB1	AAGCATGAAACGCGCTTGG	AGTTTGGCATGGTAAGTCTGC
RUNX2	TGTCATGGCGGGTAACGAT	AAGACGGTTATGGTCAAGGTGAA
ALP	CCACGTCTTCACATTTGGTG	AGACTGCGCCTGGTAGTTGT
OPN	ACTCGAACGACTCTGATGATGT	GTCAGGTCTGCGAAACTTCTTA
IBSP	CCCCACCTTTTGGGAAAACCA	TCCCCGTTCTCACTTTCATAGAT
COL1A1	CCCAAGGAAAAGAAGCACGTC	AGGTCAGCTGGATAGCGACATC
GAPDH	GGTCACCAGGGCTGCTTTTA	GGATCTCGCTCCTGGAAGATG
PPIA	GTCAACCCCACCGTGTTCTT	CTGCTGTCTTTGGGACCTTGT
U6	CTCGCTTCGGCAGCACATATACT	ACGCTTCACGAATTTGCGTGTC
FZD4	CCTCGGCTACAACGTGACC	TGCACATTGGCACATAAACAGA
siFZD4-1	CAGUAUGUGCUAUAAUAUUTT	AAUAUUAUAGCACAUACUGTT
siFZD4-2	GGCUACAACGUGACCAAGATT	UCUUGGUCACGUUGUAGCCTT
siFZD4-3	GUGCUAUGUUGGAAACCAATT	UUGGUUUCCAACAUAGCACTT

© 2018. Fan J et al. Published online at http://www.aginganddisease.org/EN/10.14336/AD.2018.1110

SUPPLEMENTARY DATA

Supplementary Table 2. The sequences of fragments in dual luciferase reporter assay

Gene name	Sequence
FZD4-WT-F	5’-TCGAGATGTAAAATGTGAACTCCCTGTGCTGCTTGTAGACAGTT CCCATAACTGTCCACGGCCCTGGAGCACGCACCCAGGGC-3’
FZD4-WT-R	5’-GGCCGCCCTGGGTGCGTGCTCCAGGGCCGTGGACAGTTATGGGA ACTGTCTACAAGCAGCACAGGGAGTTCACATTTTACATC-3’
FZD4-MUT-F	5’-TCGAGATGTAAAATGTGAACTCCCTGTGCTGCTTGTAGACATAACT GTCCACGGCCCTGGAGCACGCACCCAGGGC-3’
FZD4-MUT-R	5’-GGCCGCCCTGGGTGCGTGCTCCAGGGCCGTGGACAGTTATGTCTACA AGCAGCACAGGGAGTTCACATTTTACATC-3’
NFAT5-WT-F	5’-TCGAGCAGTGCTTTACAGTTTGAAGTGGTCACTTACCTGATGGTTCCC ACAAGCCTTAGGCTTTACAGGGTTGTATCATTGGC-3’
NFAT5-WT-R	5’-GGCCGCCAATGATACAACCCTGTAAAGCCTAAGGCTTGTGGGAACCA TCAGGTAAGTGACCACTTCAAACTGTAAAGCACTGC-3’
NFAT5-MUT-F	5’-TCGAGCAGTGCTTTACAGTTTGAAGTGGTCACTTACCTGATGCAAGCC TTAGGCTTTACAGGGTTGTATCATTGGC-3’
NFAT5-MUT-R	5’-GGCCGCCAATGATACAACCCTGTAAAGCCTAAGGCTTGCATCAGGTAA GTGACCACTTCAAACTGTAAAGCACTGC-3’
GDNF-WT-F	5’-TCGAGTTGCATTCCTGCTACAGTGCAAAGAAAGGGACCAAGGTTCCCA GGAAATGTTTGCCCAGAATGGAAGATGAGGACCGC-3’
GDNF-WT-R	5’-GGCCGCGGTCCTCATCTTCCATTCTGGGCAAACATTTCCTGGGAACCTT GGTCCCTTTCTTTGCACTGTAGCAGGAATGCAAC-3’
GDNF-MUT-F	5’-TCGAGTTGCATTCCTGCTACAGTGCAAAGAAAGGGACCAAGGGAAATG TTTGCCCAGAATGGAAGATGAGGACCGC-3’
GDNF-MUT-R	5’-GGCCGCGGTCCTCATCTTCCATTCTGGGCAAACATTTCCCTTGGTCCCTT TCTTTGCACTGTAGCAGGAATGCAAC-3’
FZD5-WT-F	5’-TCGAGTATGAAAAACAAAGAAATGTCTATTTTTCTTTGTTCCCAATTAAT GTAGATAAATTTTAAAATGCATTAAAGTAGC-3’
FZD5-WT-R	5’-GGCCGCTACTTTAATGCATTTTAAAATTTATCTACATTAATTGGGAACAA AGAAAAATAGACATTTCTTTGTTTTTCATAC-3’
FZD5-MUT-F	5’-TCGAGTATGAAAAACAAAGAAATGTCTATTTTTCTTTATTAATGTAGAT AAATTTTAAAATGCATTAAAGTAGC-3’
FZD5-MUT-R	5’-GGCCGCTACTTTAATGCATTTTAAAATTTATCTACATTAATAAAGAAAAA TAGACATTTCTTTGTTTTTCATAC-3’
CLCN5-WT-F	5’-TCGAGACTAGCCTCGAGATCTTAACCGCTCTGTGCCTCAGTGTTCCCATC TGCAAAATGGGGATAGTGTTAATTGCGC-3’
CLCN5-WT-R	5’-GGCCGCGCAATTAACACTATCCCCATTTTGCAGATGGGAACACTGAGGCA CAGAGCGGTTAAGATCTCGAGGCTAGTC-3’
CLCN5-MUT-F	5’-TCGAGACTAGCCTCGAGATCTTAACCGCTCTGTGCCTCAGTTCTGCAAA ATGGGGATAGTGTTAATTGCGC-3’
CLCN5-MUT-R	5’-GGCCGCGCAATTAACACTATCCCCATTTTGCAGAACTGAGGCACAGAG CGGTTAAGATCTCGAGGCTAGTC-3’

SUPPLEMENTARY DATA

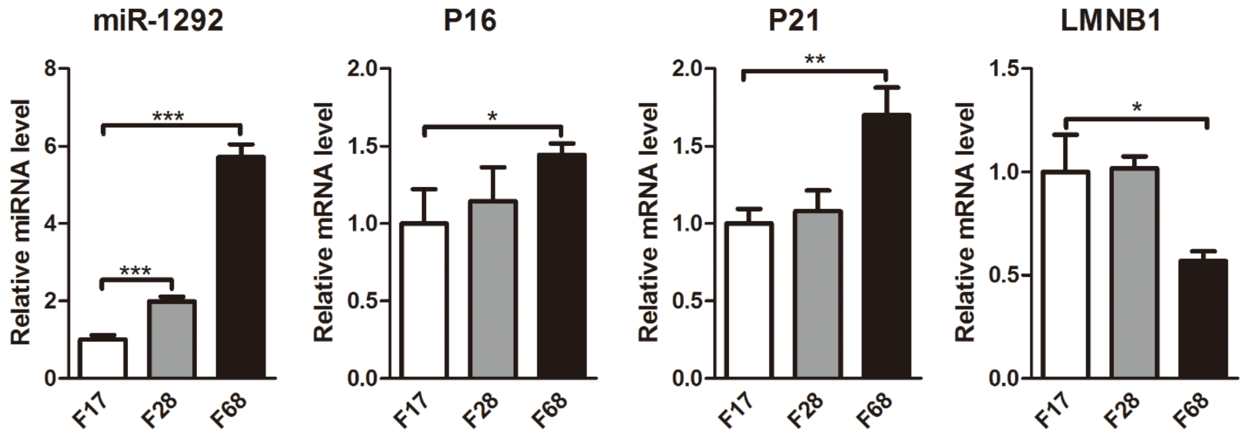

Supplementary Figure 1. miR-1292 levels increase with age in adipose tissues. qRT-PCR analysis of the expression levels of miR-1292, *P16*, *P21*, and *LMNB1* in adipose tissues obtained from females of different ages. The data, normalized to U6 or PPIA expression, are presented as the mean ± SD, n = 3. $^{*}P < 0.05$, $^{**}P < 0.01$ compared to the control

© 2018. Fan J et al. Published online at http://www.aginganddisease.org/EN/10.14336/AD.2018.1110

SUPPLEMENTARY DATA

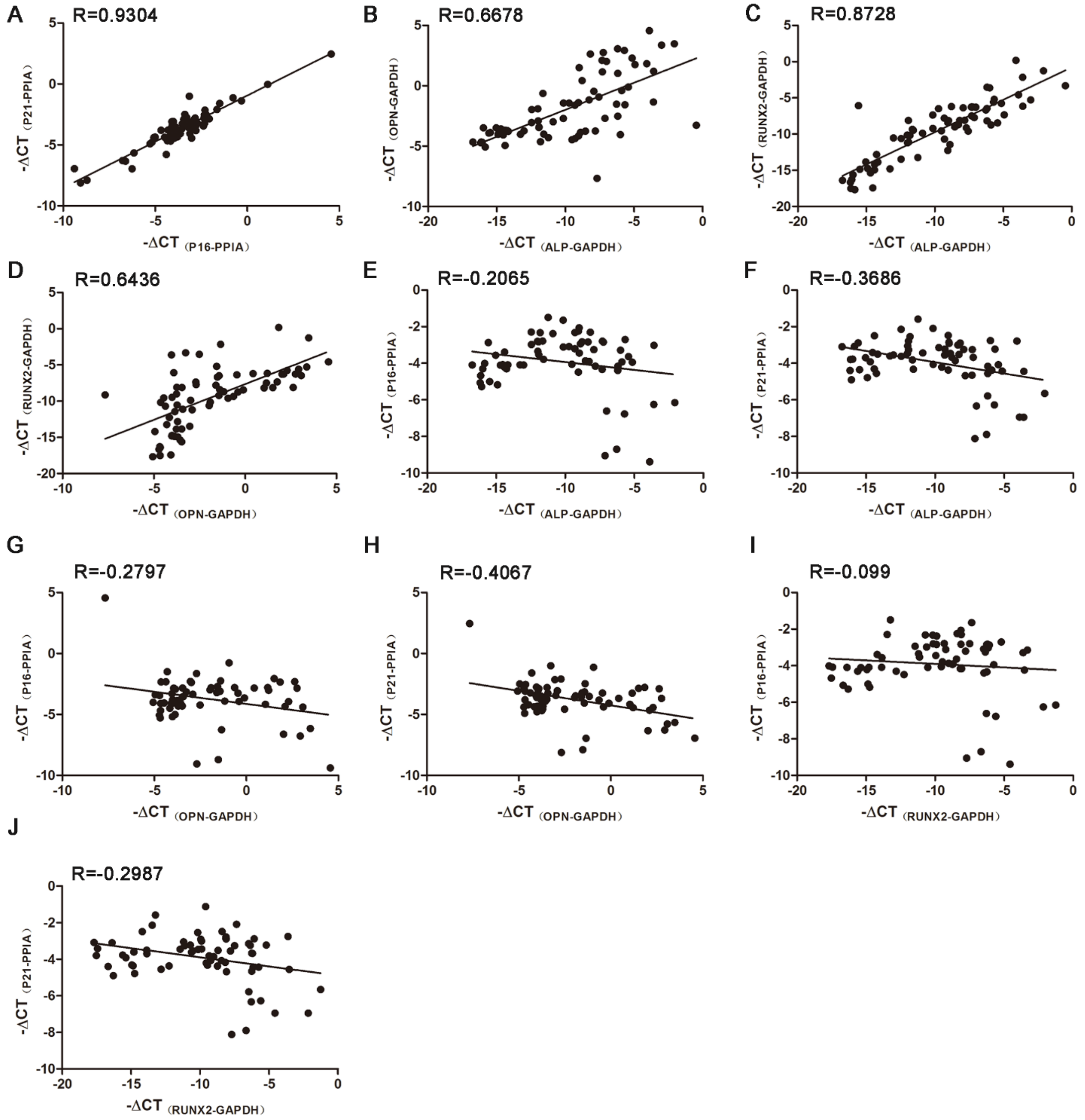

Supplementary Figure 2. Analysis of the correlation between senescence-associated markers and bone formation markers in clinical bone samples. Correlation analysis of *P16* and *P21* **(A)**, *ALP* and *OPN* **(B)**, *ALP* and *RUNX2* **(C)**, *OPN* and *RUNX2* **(D)**, *ALP* and *P16* **(E)**, *ALP* and *P21* **(F)**, *OPN* and *P16* **(G)**, *OPN* and *P21* **(H)**, *RUNX2* and *P16* **(I)**, *RUNX2* and *P21* **(D)** mRNA levels in human bone specimens. The data, normalized to PPIA or GAPDH expression, are presented as the mean ± SD, n = 3.

© 2018. Fan J et al. Published online at http://www.aginganddisease.org/EN/10.14336/AD.2018.1110

SUPPLEMENTARY DATA

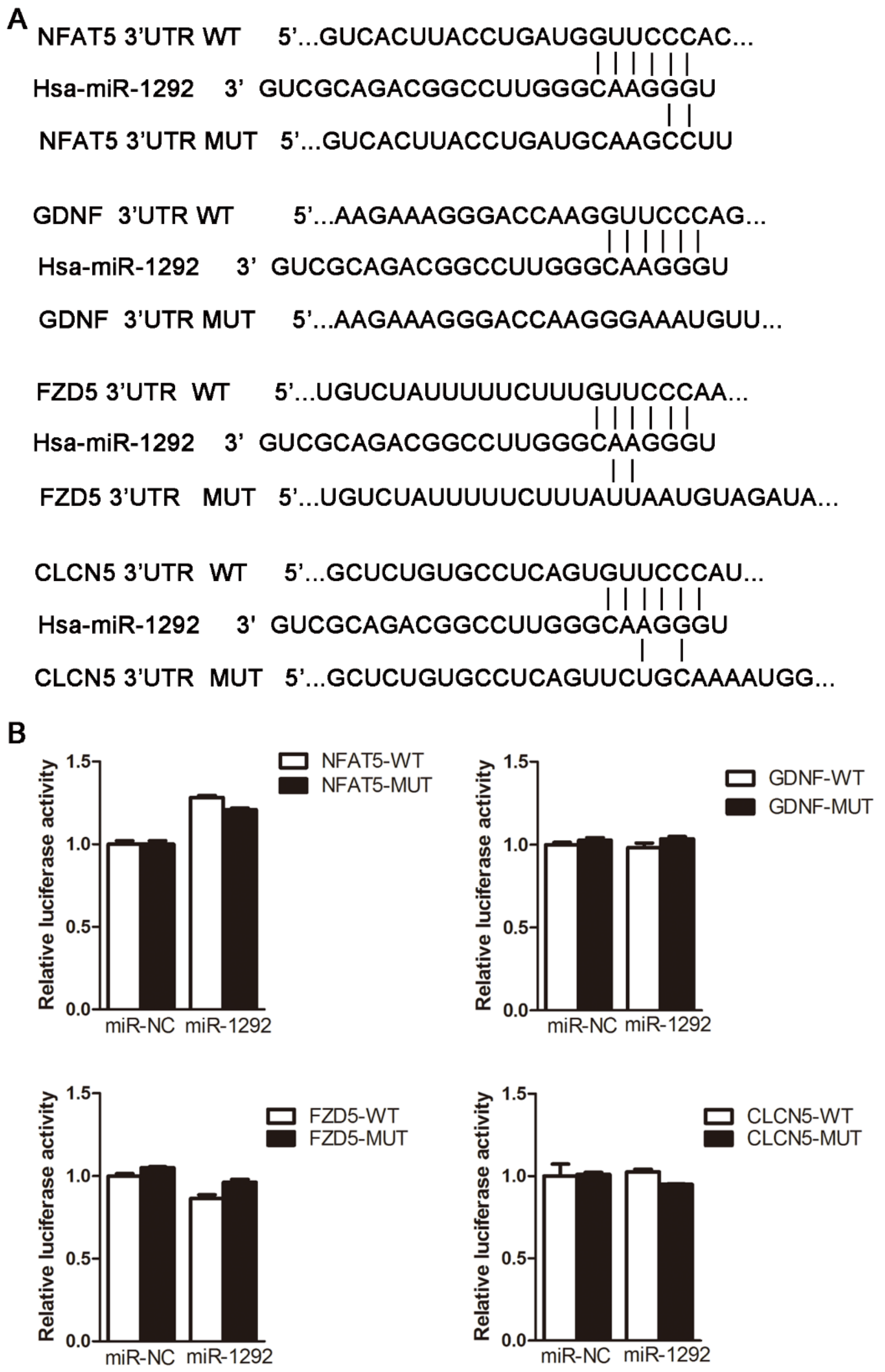

Supplementary Figure 3. ***NFAT5*, *GDNF*, *FZD5* and *CLCN5* are not direct targets of miR-1292. (A)** Computational analysis was performed to determine complementarities of the miR-1292 seed sequence to the 3′ UTR of the aforementioned genes. A wild-type (WT) or mutant-type (MUT) construct was inserted into the psiCHECK-2 reporter vector. **(B)** Luciferase activity was measured in the lysates, and values were normalized to the psiCHECK-2 vector. The data, normalized to GAPDH levels, are averages of three independent experiments (mean ± SD).

© 2018. Fan J et al. Published online at http://www.aginganddisease.org/EN/10.14336/AD.2018.1110

SUPPLEMENTARY DATA

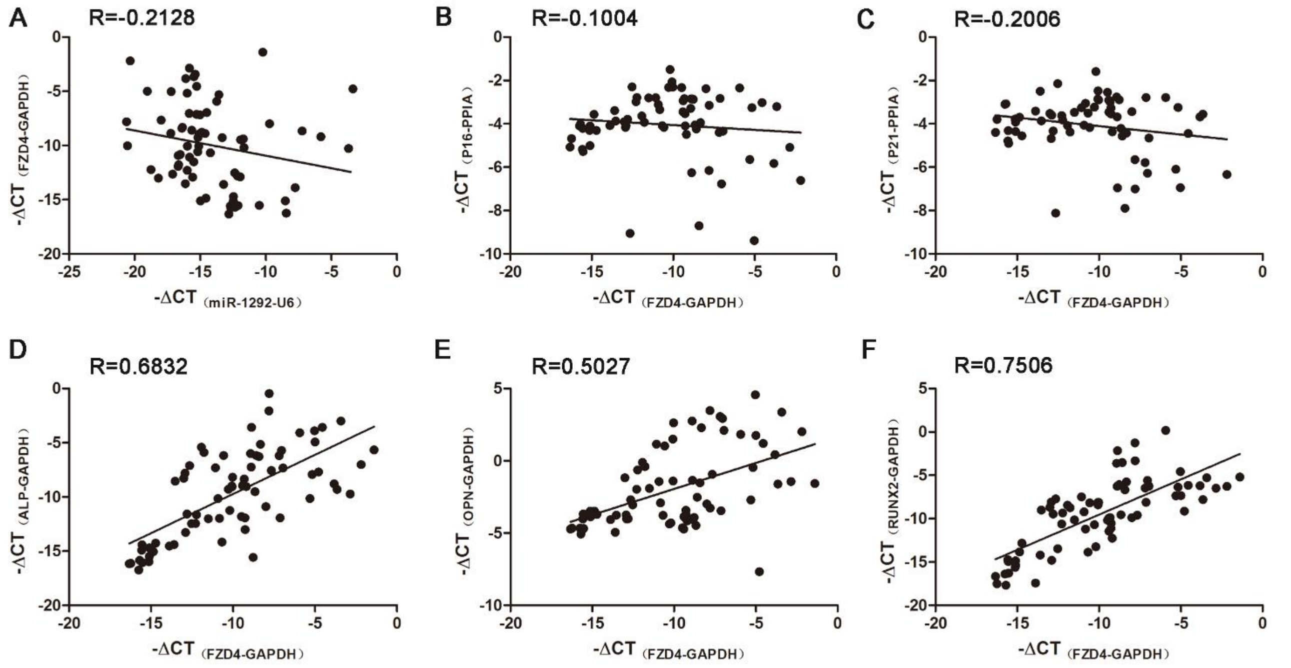

Supplementary Figure 4. Analysis of the correlations between FZD4 and miR-1292, FZD4 and senescence markers, and FZD4 and bone formation markers in clinical bone samples. Correlation analysis of miR-1292 and *FZD4* **(A)**, *FZD4* and *P16* **(B)**, *FZD4* and *P21* **(C)**, *FZD4* and *ALP* **(D)**, *FZD4* and *OPN* **(E)**, *FZD4* and *RUNX2* **(F)** mRNA levels in human bone specimens. The data, normalized to U6, PPIA or GAPDH expression, are presented as the mean ± SD, n = 3.

© 2018. Fan J et al. Published online at http://www.aginganddisease.org/EN/10.14336/AD.2018.1110
